# Surgical Resection of Metastatic Malignant Small Bowel Obstruction

**DOI:** 10.7759/cureus.27421

**Published:** 2022-07-28

**Authors:** Nickolas G Kessler, Michael Franz

**Affiliations:** 1 General Surgery, University of Central Florida College of Medicine, Orlando, USA; 2 General Surgery, Bay Pines Veterans Affairs Health Care System, St. Petersburg, USA

**Keywords:** metastasis, tumor resection, cancer, adenocarcinoma, small bowel obstruction

## Abstract

Small bowel cancer is a rare cause of small bowel obstruction (SBO) that is often discovered too late, leading to a poor prognosis at diagnosis. This case describes an African American patient with a previous history of abdominal surgery who presented to the emergency department with a partial small bowel obstruction (PSBO) that failed to resolve with conservative measures, therefore requiring surgical intervention. An exploratory laparoscopy revealed a firm apple core mass obstructing the lumen of the proximal jejunum 20 cm from the ligament of Treitz. The involved portion of the small bowel was resected with wide margins and sent to pathology. The small bowel was reconstructed by a functional end-to-end anastomosis, and the patient was admitted for observation until the return of bowel function. The pathology report, returned four weeks after the patient's discharge, reported metastatic adenocarcinoma originating from the small intestine. The patient was referred to oncology for further management of his metastatic cancer. Small bowel cancer, although rare, should always be part of the differential diagnosis in the case of small bowel obstruction. If cancer is suspected during exploratory surgery, the entire peritoneal cavity should be explored, and oncologic bowel resection should be performed with adequate margins. Final staging then occurs in the postoperative period.

## Introduction

Small bowel obstruction is a common condition often requiring surgical correction [1]. Up to 80% of obstructions occur in the small intestine, with adhesions from prior surgeries being the most common cause, followed by tumors and complicated hernias [2,3]. Of the small bowel tumors, obstruction is reported to occur in 15-29% of cases [4]. Overall, cancer of the small bowel is uncommon, occurring approximately 15 times less frequently than colon cancer in men and women annually [5]. There are several hypotheses as to why this variance exists, a few being higher rate of flow, dilution of intestinal contents, decreased bacterial concentration, and greater presence of lymphoid tissue in the small intestine [6]. Unfortunately, diagnosis of small bowel cancer is difficult due to low incidence, variability in symptomology, and lack of screening tests. This leads to delay in diagnosis, thus often higher grade of neoplasm and poorer prognosis [7]. This case describes a patient with metastatic adenocarcinoma of the proximal jejunum causing partial small bowel obstruction (PSBO).

## Case presentation

A 60-year-old African American male presented to the emergency department with a chief complaint of progressive abdominal pain for one month in addition to two weeks of constipation, nausea, vomiting, and inability to keep food down. The pain was described as diffuse cramping pain in the upper abdomen. He had never experienced these symptoms before. His last bowel movement was ten days prior and noted to be melanotic. The patient denied symptoms of fever, chills, difficulty breathing, chest pain, unintentional weight change, recent travel, or consuming any new or unusual foods. Medical history was significant for obesity and clear cell carcinoma of right kidney status post resection, without recurrence. Surgical history was significant for laparoscopic partial resection of the right kidney two years earlier for clear cell carcinoma, grade two, with negative margins. There was no family history of gastrointestinal (GI) malignancy.

On physical exam, vital signs were within normal limits, and the patient was alert, oriented, and answered questions appropriately. The patient's abdomen was distended with tenderness to palpation in the upper quadrants bilaterally. No signs of peritonitis or masses were appreciated. A few small well-healed scars were noted on the right abdomen from his previous nephrectomy. The remainder of his physical examination was unremarkable.

**Figure 1:**
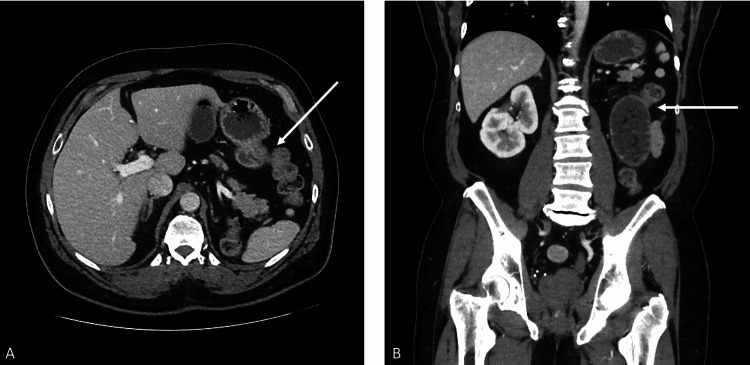
CT of the abdomen without contrast: (A) transverse plane and (B) coronal plane There are moderate recent distended loops of the small bowel from the level of the duodenal C-loop down to the left pelvic inlet. This finding has developed since a previous study and presumably represents a partial small bowel obstruction (arrows). The exact etiology and location of the obstruction are not entirely certain but might be related to adhesion. The more distal small bowel beyond this level is normal in caliber.

Significant findings on further workup included normocytic anemia, with no other abnormalities. Computed tomography (CT) of the abdomen and pelvis without contrast revealed distended loops of small bowel from the level of duodenal C-loop down to left pelvic inlet with more distal small bowel beyond this level showing normal caliber (see Figure 1). These findings were consistent with a moderate degree of PSBO.

**Figure 2:**
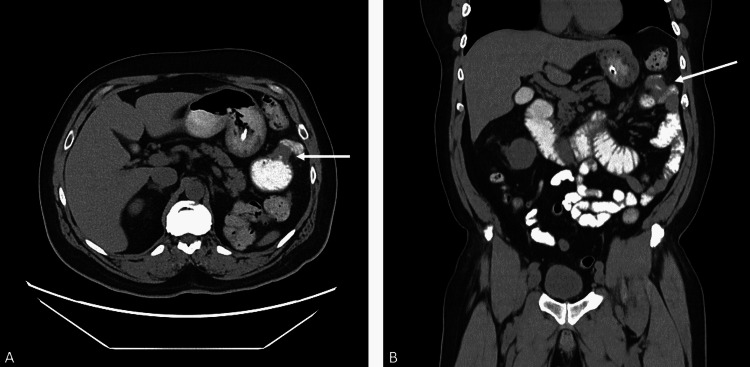
CT of the abdomen with barium sulfate contrast: (A) transverse plane and (B) coronal plane Focal narrowed segment of proximal to mid jejunum in the left upper quadrant responsible for partial bowel obstruction (arrows). Wall is thickened. The neoplastic disease is of concern, although the findings are nonspecific.

The patient was admitted to general surgery, and despite improvement of abdominal pain with nasogastric tube (NGT) decompression, he never had a bowel movement. Two days later, a repeat CT abdomen with barium sulfate contrast revealed persistence of obstruction in the proximal to mid jejunum (see Figure 2). An exploratory laparoscopy revealed near-complete small bowel obstruction at the proximal jejunum, 20 cm from the ligament of Treitz, due to a firm apple core mass. The surgery was converted to an open laparoscopic-assisted incision, and focal resection of the lesion was performed with wide margins extending into the grossly normal-appearing small intestine, mesentery, and omentum. Tissue samples were sent to pathology for diagnosis and staging. The small bowel was reconstructed using a side-to-side, functional end-to-end stapled anastomosis.

Postoperatively, the patient was maintained on "nothing by mouth" orders until the return of bowel function, which occurred on a postoperative day (POD) five. Repeat CT abdomen and pelvis with contrast confirmed good anastomotic seal without a leak. The patient's diet was advanced slowly until he tolerated full liquids. He was discharged on POD seven with a good return of bowel function, tolerating liquid diet, and pain control. The plan upon discharge was to continue soft food and liquid diet for two weeks until a follow-up appointment for reassessment. The pathology report returned indicating moderate-to-poorly differentiated metastatic small bowel adenocarcinoma with invasion into the omental and subserosa wall, involving three out of four lymph nodes. At discharge, the patient was referred to oncology and started on palliative chemotherapy.

## Discussion

Gastrointestinal obstruction is a common medical condition encountered by clinicians. Obstruction occurs most often in the small bowel usually due to adhesion, complicated hernias, or tumors [2,3]. Interestingly, cancer of the small intestine is rare, accounting for less than 5% of all GI cancers [7]. More specifically, adenocarcinoma is reported to be responsible for around 33% of malignant small bowel tumors [8]. Adenocarcinomas most often develop in the duodenum, followed by the jejunum, then the ileum [9]. The clinical presentation of small bowel adenocarcinoma is variable. The reported frequencies of presenting symptoms are as follows: abdominal pain (43%), nausea and vomiting (16%), anemia (15%), GI bleed (7%), weight loss (3%), or other non-specific symptoms (9%) [10]. In this case, the patient presented with many of these non-specific symptoms, including abdominal pain for one month, nausea and vomiting for 10 days, and normocytic anemia.

While signs of small bowel obstruction are likely to lead to an investigation to rule out small bowel cancer, the diagnosis is often missed when the patient presents with non-specific symptoms such as abdominal pain or nausea and vomiting. This is postulated to be a reason for the delay in diagnosis of small bowel cancers leading to the discovery of cancer at a more advanced stage with a poorer prognosis [7]. We believe that general practitioners should maintain a heightened index of clinical suspicion for small bowel cancer in patients presenting with vague abdominal symptoms, especially in patients with predisposing risk factors. These factors include male sex, age 60-70 years old, African American race, diets high in red meat, salted or smoked foods, history of colon cancer, inflammatory bowel disease, Celiac disease, or inherited syndromes, such as familial adenomatous polyposis or Lynch syndrome [11].

Upon clinical suspicion of small bowel cancer, patients should undergo further evaluation with either CT abdomen and pelvis with oral and IV contrast, video capsule endoscopy, or enteroclysis, which is reported to have higher sensitivity for small bowel malignancies [12]. If diagnostic findings are suspicious or even inconclusive, the patient should be referred to a surgical specialist for exploratory laparotomy. The recommended treatment of small bowel cancer includes wide surgical resection (8-10 cm margins), including the surrounding mesentery, with a goal of retrieval of eight or more regional lymph nodes in an ideal scenario, though this is not always achieved as in this case [13]. The literature suggests that the five-year survival benefit of complete resection is substantial, i.e., 42% compared to 6% in incomplete resection. However, in the case of small bowel obstruction as the presenting symptom, resection should be attempted for palliative relief, even if only partial resection is possible, regardless of the cancer stage [14]. The tumor should be sent to pathology and staged for consideration of adjunctive therapy after resection. Chemotherapy and radiation can be considered for stage III and IV disease with node-positive cancer and positive resection margin; however, there is no conclusive data on whether this improves survival rates [15].

Once the obstructing tumor is removed, the resected ends of the small bowel should be re-anastomosed. We performed a standard functional "end-to-end" anastomosis to re-establish the normal or "physiologic" gut motility [16]. Some early complications of this procedure can be anastomotic leak, abscess, infection, and bleeding. An anastomotic leak will usually present clinically within five to seven days after surgery [17]. For this reason, patients should be monitored in the hospital until the return to normal bowel function. If a rare instance of a small bowel anastomosis leak is suspected, an early post-operative GI contrast study with Gastrografin® may be performed, with a CT scan being the most sensitive and specific [18].

## Conclusions

Small bowel cancer is a rare form of gastrointestinal cancer and can present with small bowel obstruction. In a patient with non-specific abdominal symptoms, new-onset of obstruction, and a history of cancer, small bowel malignancy should be considered highly in a clinician's differential diagnosis. Abdominal imaging should be ordered, and if small bowel cancer is suspected or if symptoms fail to improve, especially without a diagnosis, then exploratory laparotomy with possible resection and anastomosis is the preferable option.

## References

[1] Cappell MS, Batke M (2008). Mechanical obstruction of the small bowel and colon. Med Clin North Am.

[2] Drożdż W, Budzyński P (2012). Change in mechanical bowel obstruction demographic and etiological patterns during the past century: observations from one health care institution. Arch Surg.

[3] Markogiannakis H, Messaris E, Dardamanis D (2007). Acute mechanical bowel obstruction: clinical presentation, etiology, management and outcome. World J Gastroenterol.

[4] Winner M, Mooney SJ, Hershman DL, Feingold DL, Allendorf JD, Wright JD, Neugut AI (2013). Incidence and predictors of bowel obstruction in elderly patients with stage IV colon cancer: a population-based cohort study. JAMA Surg.

[5] (2022). Cancer stat facts: small intestine cancer. https://seer.cancer.gov/statfacts/html/smint.html.

[6] Weiss NS, Yang CP (1987). Incidence of histologic types of cancer of the small intestine. J Natl Cancer Inst.

[7] Ciresi DL, Scholten DJ (1995). The continuing clinical dilemma of primary tumors of the small intestine. Am Surg.

[8] Bilimoria KY, Bentrem DJ, Wayne JD, Ko CY, Bennett CL, Talamonti MS (2009). Small bowel cancer in the United States: changes in epidemiology, treatment, and survival over the last 20 years. Ann Surg.

[9] Gabos S, Berkel J, Band P, Robson D, Whittaker H (1993). Small bowel cancer in western Canada. Int J Epidemiol.

[10] Halfdanarson TR, McWilliams RR, Donohue JH, Quevedo JF (2010). A single-institution experience with 491 cases of small bowel adenocarcinoma. Am J Surg.

[11] Chow WH, Linet MS, McLaughlin JK, Hsing AW, Chien HT, Blot WJ (1993). Risk factors for small intestine cancer. Cancer Causes Control.

[12] Bessette JR, Maglinte DD, Kelvin FM, Chernish SM (1989). Primary malignant tumors in the small bowel: a comparison of the small-bowel enema and conventional follow-through examination. AJR Am J Roentgenol.

[13] Wilhelm A, Müller SA, Steffen T, Schmied BM, Beutner U, Warschkow R (2016). Patients with adenocarcinoma of the small intestine with 9 or more regional lymph nodes retrieved have a higher rate of positive lymph nodes and improved survival. J Gastrointest Surg.

[14] Talamonti MS, Goetz LH, Rao S, Joehl RJ (2002). Primary cancers of the small bowel: analysis of prognostic factors and results of surgical management. Arch Surg.

[15] Meijer LL, Alberga AJ, de Bakker JK (2018). Outcomes and treatment options for duodenal adenocarcinoma: a systematic review and meta-analysis. Ann Surg Oncol.

[16] Goulder F (2012). Bowel anastomoses: the theory, the practice and the evidence base. World J Gastrointest Surg.

[17] Clatterbuck B, Moore L (2022). Small bowel resection.

[18] Nicksa GA, Dring RV, Johnson KH, Sardella WV, Vignati PV, Cohen JL (2007). Anastomotic leaks: what is the best diagnostic imaging study?. Dis Colon Rectum.

